# Habits, Quick and Easy: Perceived Complexity Moderates the Associations of Contextual Stability and Rewards With Behavioral Automaticity

**DOI:** 10.3389/fpsyg.2019.01556

**Published:** 2019-07-24

**Authors:** Kiran McCloskey, Blair T. Johnson

**Affiliations:** Institute for Collaboration on Health, Intervention, and Policy, Department of Psychological Sciences, University of Connecticut, Storrs, CT, United States

**Keywords:** automaticity, behavior, behavioral automaticity, habit, habit strength

## Abstract

**Background:**

Habits have been proposed to develop as a function of the extent to which a behavior is rewarded, performed frequently, and executed in a stable context. The present study examines how each of these factors are associated with behavioral automaticity across a broad variety of behaviors drawn from previous habits research. This study further assesses how perceived complexity of the behavior influences the associations of rewards, frequency, and contextual stability with automaticity.

**Methods:**

Participants (*N* = 459) completed an online survey assessing their experiences and engagement with 25 different behaviors, including exercise, handwashing, smoking, and medication adherence, among others. Exploratory factor analysis validated a short, relatively novel scale of perceived behavioral complexity, and multilevel analyses grouped by participant were used to examine the factors that contribute to automaticity.

**Results:**

Across behaviors, frequency, contextual stability, and perceived rewards were positively associated with automaticity. Perceived complexity was negatively associated with automaticity and moderated the influence of contextual stability and rewards, but not frequency, on automaticity. Both contextual stability and rewards were stronger predictors of automaticity when behavioral complexity was high rather than low, as predicted; in addition, when contextual stability was high, more complex behaviors showed *greater* automaticity than simpler behaviors.

**Conclusion:**

The results of this study confirm that behavioral frequency, rewards, and contextual stability are each independently associated with automaticity across a spectrum of behaviors. This study further demonstrates that perceived complexity of a behavior moderates the extent to which contextual stability and rewards are associated with automaticity. The results affirm a need to further understand the components of habits and how they differ across varying behaviors.

## Introduction

As people go through their days, they execute thousands of behaviors. Some behaviors may be complex, such as going to the gym in the morning, and other behaviors may be simple, such as shutting off the lights before one leaves the house. Some behaviors may promote health; others may harm it. As behavior has important consequences for individuals’ life outcomes, impacting numerous domains such as health, career, and relationships, a large body of literature aimed at predicting behavior has developed. Perspectives such as the Theory of Planned Behavior (TPB) posit that behavior is the direct result of intention, and thus strive to uncover the factors that motivate individuals to engage in particular behaviors (Fishbein and Ajzen, 1975). Other approaches aim to understand the automatic influences that drive behavior regardless of an individual’s intentions. One particular approach focuses on the influence of *habits.* Habits are behaviors that are performed repeatedly and with little preceding forethought (Ouellette and Wood, 1998). As about 45% of people’s behavior might qualify as habitual (Neal et al., 2006), understanding habits is an important direction for behavior research.

In psychology, habits might be understood as impulses toward a behavior that are generated automatically in response to an environmental cue from a context in which that behavior has previously been repeatedly executed (Lally and Gardner, 2013), or as the dominant responses that are mentally accessible in the presence of such an environmental cue (Wood and Neal, 2009). The concept of *habit* has been applied to predict diverse behaviors such as recycling, seafood consumption, consumer behaviors, ‘cyber loafing’ at work, use of information technology, exercise, and even negative thinking (Low, 2016). In a meta-analysis of 72 studies of exercise behavior, Hagger et al. (2002) showed that including past behavior explained 19% of the variance in later behavior over and above the variance accounted for by TPB variables. A second meta-analysis examined a broad spectrum of behaviors and found that past behavior explained additional variance after accounting for TPB variables: 3.4% for dietary behaviors, 10.3% for physical activity behaviors, 11.4% for abstinence behaviors, and 25.3% for health-risk behaviors (McEachan et al., 2011). In fact, when including past behavior in the model, past behavior was the *only* significant predictor of health-risk behaviors. Thus, understanding the mechanisms whereby past behavior predicts future behavior is key to understanding the determinants of many important behaviors.

Three major ‘ingredients’ have been proposed to be associated with habit formation: contextual stability, behavioral frequency, and rewards (Wood and Neal, 2016). Habits are environmentally linked, such that a cue in the environment automatically triggers an impulse toward a behavioral tendency (Wood, 2017). When a behavior is performed regularly in a stable context, the individual is more likely to encounter consistent cues that can form the basis for a context-behavior association. As frequency of this behavior increases, so too can the strength of the context-behavior association (Wood and Neal, 2009). Rewards – either intrinsic or extrinsic – may contribute to this process by encouraging behavioral repetition (Wood and Neal, 2009; Johnson et al., 2019), or by strengthening the ability of behavioral repetition to contribute to habit strength (de Wit and Dickinson, 2009). Previous research has examined the roles of these components individually. For instance, Verplanken (2006) established that, while behavioral frequency contributed to habits, behavioral frequency alone cannot explain the full impact of habits. Meanwhile, Wood et al. (2005) demonstrated that changing contexts disrupted habits. Indeed, the associations of frequency and contextual stability with habit strength are so well accepted that the multiplicative interaction of behavioral frequency and contextual stability (BF × CS) has been often used as a measurement of habit strength (see Ouellette and Wood, 1998). Phillips et al. (2016) have also shown that intrinsic rewards predict exercise behavior through intentions for those beginning an exercise routine, but through habit strength for those maintaining a previous routine. A further, recent study found that intrinsic motivation and pleasure strengthened the repetition-habit association for new behaviors (Judah et al., 2018). Yet, to date, no single study has simultaneously mapped the relative weights of each of these three components (frequency, contextual stability, and reward) in their associations with automaticity. Further, there has been no research assessing how each of these components contribute to automaticity across a spectrum of behaviors.

As mentioned, McEachan et al. (2011) found that different types of behavior were differentially predicted by past behavior; therefore, there is a need to understand how characteristics of behaviors influence automaticity. The *complexity* of the behavior has been proposed to impact the development of habit-related automaticity (Wood et al., 2002; Verplanken, 2006; Wood and Neal, 2009; Lally et al., 2010). Behavioral complexity can be understood as the number of physical or mental steps involved in executing the behavior, in which behaviors that are complex are more time-consuming and require a greater amount of planning; for example, simple behaviors are exemplified by handwashing or cigarette smoking and complex behaviors by performing well on an intellectual task or quitting smoking (Boynton, 2005). More complex behaviors may have reduced habit strength compared to simple behaviors due to the number of steps that must be learned before the behavior becomes automatic. Verplanken (2006) showed that when behavioral complexity was experimentally manipulated in a laboratory word-search task, habit formation was impeded, even when frequency was kept constant. In a daily diary study, Wood et al. (2002) further found that greater complexity of a task was associated with more thoughts about the task, which may indicate that simpler tasks are more automatic. Further generalization of this association to a broad spectrum of behaviors can bolster these findings, and other measures can assess the influence of complexity as perceived by the individual doing the behavior.

Behavioral complexity may also moderate the associations of frequency, contextual stability, and rewards with behavioral automaticity, but these interactions have not yet been tested. We developed several hypotheses *a priori* and listed them in our institutional review board protocol, along with rationales for each (although we did not pre-register them otherwise). Specifically, behavioral frequency might be a stronger predictor of automaticity of simple behaviors, rather than complex behaviors, due to the number of steps that need to be learned in complex behaviors. Indeed, in the previous study by Verplanken (2006), habit strength for a novel behavior depended on complexity when behavioral frequency was kept constant. If habit strength presumably began at equal points (i.e., no habit strength) for each of these novel simple and complex behaviors, the differential development of habit strength over repeated actions would imply an interaction effect between frequency and complexity. Specifically, habit strength developed more slowly over repetition when the behavior was complex, rather than when it was simple. Yet, this previous study did not directly test an interaction between frequency and contextual stability. The present study examines such an interaction.

Conversely, contextual stability may be a weaker predictor of automaticity for simple behaviors compared to complex behaviors. Whereas the habits literature has focused primarily on behaviors that are executed automatically in a *singular* context, other behavior literature has also considered behaviors that are cued in multiple contexts. The addiction literature, for example has shown that multiple environmental cues can yield increased craving and engaging in a problem behavior for a particular individual (Fatseas et al., 2015). Implementation intention research has also assessed the use of multiple cue-behavior associations, but demonstrated that developing multiple “if [cue], then [behavior]” plans does not yield effective behavioral changes, compared to setting a single if-then plan (de Vet et al., 2011; Verhoeven et al., 2013). As implementation intentions as well are thought to yield behavior by increasing cognitive accessibility of cue and behavior (Webb and Sheeran, 2008), there is need to understand the conditions under which single or multiple cues yield inclinations toward behavior. Behavioral complexity may be a factor in the association between cues and the resulting behavior, as simple behaviors might easily be performed frequently in a broad variety of contexts such that many diverse cues can become strongly associated with the behavior. A jogging habit, for instance, may be cued only once a day when a person arrives home from work, as finding the time and planning resources to go jogging frequently at multiple times during the day would be difficult. The same individual may be cued to check their phone while making coffee, while in the bathroom, and during their lunch break. The contextual variability of this simpler behavior does not disprove its automaticity or cue-behavior associations.

Complexity may also moderate the influence of rewards on behavioral automaticity. It has been argued that rewards yield habit development through increased repetition, particularly by increasing intention to re-engage in that behavior (Rothman et al., 2009; Johnson et al., 2019). Yet, in a survey assessing individuals’ engagement with 48 different behaviors, from handwashing to seatbelt use to quitting smoking, Boynton (2005) also showed that intention is a stronger predictor of engagement in behavior when behaviors are complex, rather than when they are simple. Thus, if both patterns appear, then it follows that rewards are likely to be stronger predictors of automaticity for complex behaviors rather than simple behaviors.

In order to examine the associations between behavioral frequency, contextual stability, rewards, and behavioral complexity on automaticity, this study utilizes and assesses three relatively new scales. Low (2016) developed one to assess contextual stability, and another to measure perceived rewards. Both scales can be easily adapted to different behaviors, but neither scale has undergone rigorous validation. Boynton (2005) developed and validated a similarly generalizable self-report scale measuring perceived behavioral complexity, but no subsequent research has replicated it. Moreover, of these three novel scales, none have been yet published in the scientific literature.

Low’s (2016) contextual stability scale drew on TPB literature to create a broader measure of what constitutes a behavioral context. Specifically, Ajzen and Fishbein’s (2005) Principle of Compatibility is the principle that predictors such as attitudes and intentions best predict behavior when they match on the behavioral elements of target, action, context, and time (TACT). Given the learned, associative nature of habits, an impulse toward a behavior is likely to be greatest when an individual encounters a situation that matches on TACT to a previous situation in which that individual has been rewarded for the behavior. Indeed, Low (2016) argued that habits’ strong predictive validity with future behavior may be in part due to the greater inherent TACT compatibility between past and future behavior. That said, while habit research has tended to examine the extent to which an individual repeats a given behavior, thus keeping constant ‘target’ and ‘action,’ context has been assessed primarily as the extent to which an individual engages in a behavior in the same place (e.g., Norman and Cooper, 2011) or in the presence of a single, researcher-generated cue (Ouellette and Wood, 1998). ‘Context,’ or the environment in which an individual engages in a behavior, could be considered in broader terms, and may also include other individuals present or the tools with which one performs the behavior (Ajzen, 1988, 2002). A pianist cannot play music unless an instrument is present, for example, and the presence of an electronic keyboard, compared to the presence of a piano, may afford different behavioral impulses. Low’s measure, drawing on the Principle of Compatibility, includes the social context, tools, and manner with which the behavior is performed.

Previous published research assessing rewards in habit strength have measured reward constructs with a single item (e.g., Wiedemann et al., 2014; Judah et al., 2018), or through behavior-specific scales assessing intrinsic motivation to engage in a behavior (e.g., Phillips et al., 2016). Low’s measure of rewards assesses the emotional and physical feelings of engaging in a behavior, as well as the feelings of *not* engaging in that behavior, and examines both positive and negative feelings. As a result, Low’s scale potentially affords a more expansive and broadly applicable measure than is presently available.

Behavioral complexity has been assessed in previous habits literature, either through experimental manipulation (e.g., Verplanken, 2006) or through judgment on the part of the researcher (e.g., Wood et al., 2002; Lally et al., 2010). To our knowledge, Boynton’s (2005) scale represents the only validated self-report survey of individuals’ perceptions of behavioral complexity; her study found that this scale has good reliability and construct validity across 48 different behaviors. The present study aims to replicate these findings with our selection of 25 behaviors, including health behaviors and behaviors more contemporarily relevant to current lifestyles (e.g., mobile phone checking). Use of a measure of *perceived* behavioral complexity also has potential value for the literature, as perception of behavioral barriers do not always correlate with objective measures of such behaviors (McGinn et al., 2007), but perception of difficulty nevertheless has the potential to influence behavior (Gilpin et al., 2004).

By measuring the influence of behavioral frequency, contextual stability, and rewards on automaticity across a spectrum of 25 different behaviors, the present study examines the ‘ingredients’ of habit development proposed by Wood and Neal (2016) to draw together the wide reaches of the habits literature – from exercise behavior to negative thinking. In addition, the present study expands on the tools available for examining habitual processes by testing the psychometric characteristics of three scales related to theorized components of habits, and furthers the discussion of habits by considering how characteristics of the behavior (complexity) contribute to automaticity.

## Materials and Methods

### Participants and Procedure

Participants were recruited using MTurk; they were required to be 18 or older and to reside in the United States. After reviewing an information sheet and indicating agreement with the procedures, participants were directed to complete a survey using Qualtrics. Each participant was randomized to one of three clusters in which they rated 11 behaviors on several dimensions; seven behaviors were unique in each cluster, and four behaviors (exercise, smoking, handwashing, and medication adherence) were held constant across clusters. In total, 462 surveys were returned. Three participants submitted duplicate surveys; second surveys completed by the same participant were deleted. No other surveys were removed, making for a total of 459 surveys retained for analysis (154 in the first behavior group, 152 in the second group, and 153 in the third group). Ratings were extracted only from behaviors that participants had performed, making for a total of 3,790 behavior observations. Participants were paid $5 for completing the survey.

#### Ethical Considerations

The protocol for this study was approved by the University of Connecticut Institutional Review Board on August 9th, 2018 (protocol #X18-095, available from authors on request). Potential participants were informed regarding the procedures and demands of the study prior to starting the survey, and were encouraged to contact the researchers if they had any concerns. Individuals who agreed to the demands of the study were directed to then complete the survey. Written consent was not collected; the survey was designed to be anonymous and low-risk, and obtaining signed consent would result in the collection of identifying information. A waiver of signed consent was granted by the University of Connecticut Institutional Review Board.

### Measures

#### Behavior Level (Level-1) Variables

##### Behaviors

In total, this study collected ratings on 25 different behaviors (see Appendix). For each behavior, participants first were presented with a qualifier question; participants rated the extent to which they engaged in each behavior on a 7-point Likert scale. If participants responded that they did “not at all” engage in a particular behavior, then they were directed to provide ratings only on their perceived complexity of the behavior, and their ratings were not retained for analysis in this study. All participants were presented with questions for exercise, handwashing, smoking, and medication adherence. Exercise and handwashing were chosen to act as controls across groups. Smoking and medication adherence ratings were collected from all participants to achieve power with these behaviors as the authors reasoned that most participants would neither smoke nor take medications regularly and thus, a sizeable number of participants would not be able to provide ratings about their experiences with these behaviors.

In addition to the four behaviors presented to all participants, in cluster one, participants also provided ratings on active commuting, information technology use, sunscreen use, sitting, flossing, recycling, and playing music (either by singing or playing an instrument). In cluster two, participants also provided ratings on car use, making savings deposits, condom use, negative self-thoughts, sugary drink consumption, checking their phone, and texting and driving. In cluster three, participants also provided ratings on fruit and vegetable consumption, unhealthy snacking, alcohol consumption, internet use, seafood consumption, use of food safety practices, and playing video games. These behaviors were selected to represent many behaviors that have been assessed using habits in past research, as identified in a recent meta-analysis (Low, 2016).

##### Behavioral frequency

Behavioral frequency was measured with a single item. Participants who reported that they did engage in the given behavior on the qualifier question used a sliding scale to indicate how many times they engaged in that behavior in the average week, from 0 to 20 (or more) times a week.

##### Contextual stability

Contextual stability was assessed using the eight items Low (2016) developed to assess contextual stability of a behavior based on the factors of Ajzen and Fishbein’s (2005) Principle of Compatibility. Each item in this scale was scored on a scale from 0 to 10.

##### Perceived rewards

Perceived rewards were assessed as the feelings elicited by doing a behavior, using the items Low (2016) developed. This scale includes six items that assess the physical and emotional feelings individuals experience as a result of doing or not doing a particular behavior, and assesses both good and bad feelings. Each item in Low’s scale is scored from 0 to 10.

##### Perceived behavioral complexity

Perceived behavioral complexity was measured with the six-item scale that Boynton (2005) developed and validated. This scale assesses the perceived steps involved in executing a particular behavior by measuring the extent to which an individual views a particular behavior as difficult, time-consuming, and requiring significant planning for the average adult. Each item was assessed on a 7-point Likert scale.

##### TPB components

Perceived behavioral control and intention were measured based on the guidelines Fishbein and Ajzen (2011) provided. *Perceived behavioral control* was measured using two 7-point Likert items: “I am confident I am capable of [doing behavior],” and “whether or not I [do behavior] is up to me.” *Behavioral intention* was measured with a single 7-point Likert item: “I intend to engage in this behavior.” For the purposes of this analysis, we included only TPB components that have been theorized to predict behavior directly. (The TPB variables of attitude and social norm were also measured but not analyzed for the present study.)

##### Automaticity

Automaticity was measured using the Self-Report Behavioral Automaticity Index (SRBAI: Gardner et al., 2012). While automaticity alone does not necessarily assess solely habits, this measure has been shown to be reliable and valid, and available is an adequate shorter version of the widely used Self Report Habit Index (SRHI: Verplanken and Orbell, 2003; Gardner et al., 2012). The measure has been applied to a wide variety of behavioral domains including safe food handling, fruit consumption, and physical activity (Low, 2016). Each item is scored on a 7-point Likert scale (from low to high).

#### Participant Level (Level-2) Variables

##### Demographics

Participants provided their gender, range of annual income, and age range. Participants also reported if they had found the survey through an online forum such as Reddit. Personality traits of conscientiousness and neuroticism were also measured, but not reported, for the present study.

### Preliminary Analyses

Factor analyses were used to test scale validity. Exploratory factor analysis was applied to the three relatively new scales used in this study: behavioral complexity, contextual stability, and rewards. Confirmatory factor analyses were used to test the validity of the scales that have been previously well-supported. Exploratory factor analysis was run in SPSS version 25.0 (Ibm Corp., 2017). Confirmatory factor analysis was run in R (R Core Team, 2018) using the lavaan package (Rosseel, 2012). Further, intraclass correlations (ICC) were also calculated for each Level-1 variable (using adjusted scales, if deemed appropriate; see Results) to assess the extent to which the different behaviors and participants accounted for variation for each scale. Within-group ICC values, clustered by participant, were also computed between Level-1 variables using the psych package in R (Revelle, 2018).

### Main Analyses (and POMP-Scored Variables)

In order to account for the multiple behavior observations taken from each participant, multilevel models were used, in which behavior ratings were nested within participants. All multilevel models were run in R using the lme4 package (Bates et al., 2015). Level-1 predictors consisted of individual ratings of behavior, including behavioral frequency, contextual stability, rewards, and complexity of the behavior. Level-2 predictors consisted of participant-level characteristics, including age and gender. Predictors were uncentered and were entered in the model in the form of percent of maximum possible (POMP) scores, such that the intercept represented the lowest score possible for each predictor (Cohen et al., 1999). Cohen et al. (1999) recommend use of POMP scores as more intuitive than presenting varying scales with unique and often meaningless units. POMP scoring has previously been used to compare across disparate scales, most frequently in meta-analysis (Cerasoli et al., 2014). In the present study, POMP scoring eases visual comparison of variables across multiple scales. Further, POMP scoring facilitates multilevel modeling and interpretation of results, as it ensures all variables are entered in the model on equivalent scales. Gender was dummy-coded. All multilevel models included random effects of behavior and participant. Significant interactions were inspected with the jtools package in R (Long, 2018). *Post hoc* mediation analyses were run using the mediation package in R (Tingley et al., 2014). Two primary models were run.

#### Model 1

**FIGURE 1:**
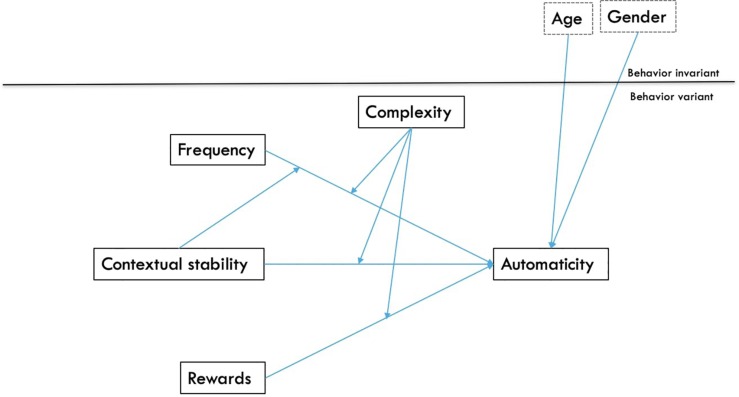
Model 1: The influence of each frequency, contextual stability, and rewards on automaticity, as moderated by complexity (conceptual model). Level 1 variables are behavior variant, meaning that within participants, multiple scores were collected for different behaviors; behavior invariant variables include Level 2 variables that represent participant characteristics that are consistent across multiple observations for different behaviors.

Model 1 tested how Level-1 variables of each behavioral frequency, contextual stability, rewards, and complexity impact automaticity, as well as how complexity interacts with the other three variables to predict automaticity. An interaction between frequency and contextual stability was also included, in order to account for the association between automaticity and the popular BF × CS measurement of habit strength. Gender and age were included as Level-2 covariates; first, main effects only were tested (reported as Model 1a), after which interactive effects were added to the model (reported as Model 1b) so as to yield accurate estimates of main and interactive effects. The model was tested with and without the interaction between frequency and contextual stability; results did not meaningfully differ, and only the model including the interaction is reported. The conceptual model appears in Figure 1. The general form of the model is given by:

$$\begin{array}{c} \mathrm{AUTO}=[\gamma_{00}+\gamma_{01}\mathrm{GENDER}+\gamma_{02}\mathrm{AGE}+\gamma_{10}\mathrm{FREQ}\\ \;+\gamma_{20}\mathrm{CONTEXT}+\gamma_{30}\mathrm{REWARD}+\gamma_{40}\mathrm{COMPLEX}\\ \;+\gamma_{50}\mathrm{COMPLEX}\times \mathrm{FREQ}+\gamma_{60}\mathrm{COMPLEX}\\ \;\times \mathrm{CONTEXT}+\gamma_{70}\mathrm{COMPLEX}\times \mathrm{REWARD}\\ \;+\gamma_{80}\mathrm{FREQ}\times \mathrm{CONTEXT}]+\varepsilon \end{array}$$

Model 1 was first run as a multilevel model across behaviors, and then again individually as a regression for each of the four behaviors presented to all participants (exercise, handwashing, smoking, and medication adherence). By re-examining Model 1 for individual behaviors, extraneous confounds introduced by assessing varying behaviors in the multilevel model (such as behavioral desirability or healthiness of the behavior) were controlled for. In particular, *objective* complexity was held constant in each individual behavior model and thus the role of *perceived* complexity was central.

#### Model 2

**FIGURE 2:**
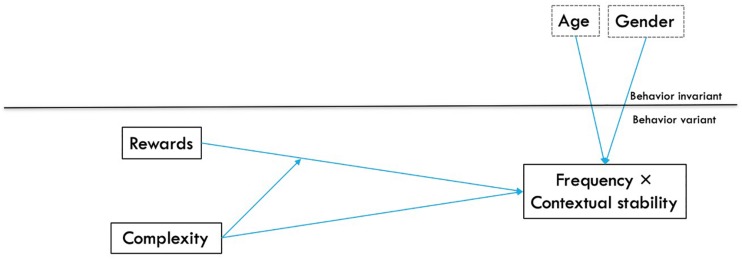
Model 2: The influence of rewards and complexity and their interaction on habit strength, as measured by frequency and contextual stability. Level 1 variables are behavior variant, meaning that within participants, multiple scores were collected for different behaviors; behavior invariant variables include Level 2 variables that represent participant characteristics that are consistent across multiple observations for different behaviors.

Model 2 aimed to replicate findings of Model 1 by testing the influence of rewards and complexity on habit strength, using the BF × CS interaction as a measure of habit strength. Age and gender were again included as Level-2 covariates, and a complexity × reward interaction was entered after main effects. The conceptual model appears in Figure 2. The general form of the model is given by:

BF×CS=[γ+00γGENDER01+γAGE02+γREWARD10+γCOMPLEX20+γCOMPLEX30×REWARD]+ε

## Results

Each participant provided ratings for an average of eight different behaviors, and each behavior was rated by an average of 152 participants (Table 1). Of all behaviors assessed in this study, handwashing was rated by the greatest number of participants (453), and texting and driving was rated by the fewest number of participants (45, representing 30% of participants presented with this behavior). Table 2 provides descriptive statistics for both Level-1 and Level-2 variables, aggregated across behaviors. The recruited sample had similar demographic characteristics to a typical MTurk sample (Huff and Tingley, 2015). Of the 459 participants, 260 (57%) participants were male, and 197 (43%) participants were female. A plurality (48%) of participants was between 25 and 34 years of age. Demographic information is available in the Supplementary Materials.

**TABLE 1:** Behaviors rated, ordered from most frequent to least frequently rated behaviors, along with means on key study variables.

**Behavior**	***N* Ratings**	**% Ratings possible**	**Frequency**	**Contextual stability**	**Rewards**	**Complexity**	**Habit strength**	**PBC**	**Intention**
Handwashing	453	98.69	89.56	61.73	57.88	32.01	78.80	94.37	93.47
Exercise	374	81.48	26.91	62.91	64.06	65.64	40.87	89.38	85.98
Medication use	257	55.99	40.19	70.62	41.17	48.04	55.38	85.13	82.82
Fruit and vegetable consumption	153	100.00	53.73	59.00	72.75	43.79	51.70	93.42	91.13
Internet use	153	100.00	91.08	68.89	73.59	48.96	76.14	92.67	94.30
IT use	152	98.70	85.20	64.02	60.86	58.46	74.25	86.70	90.98
Sitting	152	98.70	92.66	68.50	61.71	28.03	85.48	87.03	79.89
Food safety practices	149	97.39	66.54	65.75	54.83	54.19	76.68	91.95	93.10
Phone checking	147	96.71	88.03	58.70	52.18	32.65	76.43	88.97	83.48
Smoking	143	31.15	68.25	66.42	65.94	46.77	69.26	80.42	72.13
Unhealthy snacking	139	90.85	30.76	48.17	69.21	32.33	51.82	87.62	63.72
Car use	136	89.47	50.51	63.88	53.82	59.33	58.27	87.29	86.97
Recycling	133	86.36	47.18	59.13	57.82	45.69	65.79	90.76	88.72
Playing video games	125	81.70	38.72	64.19	76.88	64.43	45.97	90.80	83.65
Flossing	122	79.22	37.91	68.73	48.11	43.51	55.15	91.74	87.70
Seafood consumption	119	78.29	17.90	48.36	73.70	42.26	34.15	89.20	77.79
Negative self-thoughts	119	77.78	38.11	30.48	13.45	44.44	69.33	67.11	33.73
Depositing savings	118	77.63	13.69	57.42	72.72	53.24	48.27	85.53	88.74
Sunscreen use	113	73.38	20.49	54.70	46.37	41.14	46.62	91.40	82.81
Sugary drink consumption	110	72.37	33.41	52.69	65.73	32.94	47.34	85.32	59.61
Active commuting	109	70.78	40.32	70.60	49.08	58.22	61.37	81.59	81.26
Alcohol consumption	97	63.40	21.55	60.48	70.31	42.39	36.49	87.26	67.75
Playing music	94	61.04	42.45	54.73	80.53	61.23	58.97	84.19	82.07
Condom use	78	51.32	18.97	54.31	58.72	44.32	57.83	85.71	80.95
Texting and driving	45	29.61	28.67	46.92	36.67	64.83	49.37	72.06	51.43

*Variables are represented in the form of percent of maximum possible (POMP) scores so that higher scores represent more of the variable, using the adjusted scales where applicable (see preliminary results for more details). PBC, Perceived behavioral control. See Appendix for detailed definitions of each behavior.*

**TABLE 2:** Descriptive statistics for within-person (Level 1) variables.

	***M***	***SD***
**Habit variables**		
Automaticity	60.41	29.84
Behavioral complexity	46.90	23.93
Contextual stability	60.72	21.88
Rewards	59.25	30.44
Frequency	52.02	36.85
**Theory of Planned Behavior variables**		
Perceived behavioral control	87.99	15.46
Intention	82.23	22.93

*These descriptive statistics are drawn from the percent of maximum possible (POMP) scores, using the adjusted scales where applicable (see preliminary results for more details).*

### Preliminary Analyses

#### Missing Data

In total, 375 items were missing (0.0019% of items possible). The key dependent variable of automaticity was determined to be non-normally distributed using a Shapiro–Wilk normality test (*W* = 0.90, *p* < 0.001), and thus imputation was performed in R with the MICE package (van Buuren and Groothuis-Oudshoorn, 2011) using predictive means matching, which is particularly appropriate for non-normal data (Morris et al., 2014). Mean differences between the imputed and non-imputed datasets were assessed for each item (Diggle et al., 1995; Dong and Peng, 2013), and no significant differences were found for any items.

#### Differences Between Groups

There were no significant differences for behavior group for age [*F*(2,456) = 2.83, *p* = 0.060] or for gender [for being male, *F*(2,456) = 3.014, *p* = 0.050; for being female, *F*(2,456) = 2.89, *p* = 0.056; two participants selected ‘other’ as their gender]. Nonetheless, as these analyses approached significance, age and gender were retained as covariates for further analyses.

#### Scale Reliability and Validity

Of the scales used in this analysis, all but the scale for rewards had acceptable reliability. Contextual stability showed a reliability of α = 0.85, 95% *CI* [0.85, 0.86] (ranging from α = 0.77 to α = 0.93 for individual behaviors); behavioral complexity had a reliability of α = 0.84, 95% *CI* [0.84, 0.85] (ranging from α = 0.55 to α = 0.91 for individual behaviors). One item on this scale consistently reduced the reliability of the complexity scale (“For the average adult, how automatic is this behavior?”); this item was further inspected in factor analysis and ultimately removed for multilevel analysis. Without this item, the behavioral complexity scale had a reliability of α = 0.92 (ranging from α = 0.77 to α = 0.96 for individual behaviors). The SRBAI had consistently high reliability (α = 0.96, 95% *CI* [0.96, 0.96], ranging from α = 0.90 to 0.97 for individual behaviors).

The scale for rewards had a poor reliability of α = 0.51, 95% *CI* [0.49, 0.54] (ranging from α = 0.03 to α = 0.69 for individual behaviors). Exploratory factor analyses on the underperforming rewards scale suggested two factors, but the scale fit poorly onto two factors (RMSEA = 0.69, 95% *CI* [0.67, 0.71]). Given the poor reliability and validity of the rewards scale, main analyses were performed using only a single item from this scale (“When you [do behavior], how pleasurable does it feel?”). This approach is in line with previous research that has associated pleasure with habit strength (Judah et al., 2018).

Exploratory factor analysis for the behavioral complexity scale also suggested two factors, but the scale did not fit well on a two-factor model (RMSEA = 0.20, 95% *CI* [0.18, 0.22]); item analysis revealed that the second factor was driven entirely by a single item (“For the average adult, how automatic is this behavior?”). As this item also reduced the overall reliability of the scale and was determined to be particularly similar to our dependent variable of automaticity, the item was removed; when removed, the complexity scale fit well onto a single factor (RMSEA = 0.045, 95% *CI* [0.035,0.059]). Thus, further analyses were completed using the five-item version of the complexity scale. For contextual stability, exploratory factor analysis also suggested two factors. Item analysis suggested the two factors represented a factor of stability of the physical environment, and a factor of stability of the social environment. Yet, the scale did not optimally fit onto a two-factor model (RMSEA = 0.24, 95% *CI* [0.24, 0.25]). Further, despite good reliability of the scales, the measure for contextual stability also did not map well onto a single factor (RMSEA = 0.18, 95% *CI* [0.17, 0.18]). Removing the two items that loaded on the social environment factor did not improve the fit of this scale, and thus the full scale was retained. The SRBAI showed acceptable fit for a one-factor model (RMSEA = 0.072, 95% *CI* [0.054, 0.092]). The Appendix shows all scales as used for analysis.

#### Intraclass Correlations

First, empty multilevel linear models with random effects of *behavior* were used to compute an ICC for each Level-1 variable. As frequency and automaticity were found to be bimodally distributed around the extremes, these variables were stratified into ‘low’ and ‘high’ using a median split, and a logistic multilevel regression was run to compute ICC scores, using the formula proposed by Zeger et al. (1988). Frequency had an ICC of 0.48; automaticity had an ICC of 0.21. With a Gaussian distribution, contextual stability showed an ICC of 0.16, rewards showed an ICC of 0.22, and behavioral complexity had an ICC of 0.22. In addition, ICC values were also calculated using empty multilevel linear models with random effects of *participant*. With random effects of participant, rewards had an ICC of 0.29, contextual stability 0.36, and behavioral complexity 0.22. Using logistic models, frequency showed an ICC of 0.08 and automaticity 0.27 with random effects of participant. Within-group ICC values between Level-1 variables, clustered by participant, are reported in Table 3.

**TABLE 3:** Within-group intraclass correlations (ICC) values between Level-1 variables, clustered by participant (all *p*s < 0.001).

**Measure**	**1**	**2**	**3**	**4**	**5**	**6**	**7**
(1) Automaticity	1.00						
(2) Frequency	0.65	1.00					
(3) Contextual stability	0.30	0.31	1.00				
(4) Reward	0.10	0.13	0.18	1.00			
(5) Complexity	–0.26	–0.25	0.06	0.05	1.00		
(6) Perceived behavioral control	0.12	0.18	0.26	0.26	–0.07	1.00	
(7) Intention	0.27	0.34	0.45	0.34	0.07	0.44	1.00

### Main Analyses

#### Model 1

**FIGURE 3:**
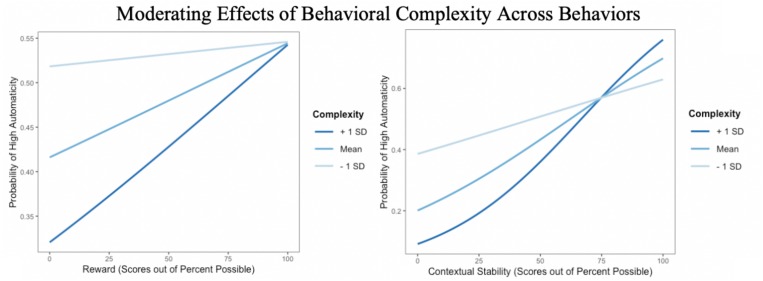
**(Left)** Probability of high automaticity across behaviors as a function of the reward value of the behavior, moderated by behavioral complexity. **(Right)** Probability of high automaticity across behaviors as a function of the stability of the context in which one does the behavior, moderated by behavioral complexity; lines curve due to the logistic analysis.

Model 1 (Figure 1) was conducted using a multilevel generalized linear model with a binomial logistic distribution, due to the non-normal distribution of automaticity. Model 1a tested main effects and found frequency, contextual stability, and rewards positively predicted behavioral automaticity, while behavioral complexity and age negatively predicted automaticity. Model 1b also included interactive effects; two significant interactions appeared (Table 4). At high levels of behavioral complexity, as hypothesized, rewards were more predictive of high automaticity compared to at low levels of behavioral complexity (Figure 3, left panel). Complexity interacted with contextual stability as predicted such that when behaviors were perceived as complex, contextual stability was a stronger predictor of high behavioral automaticity than when behaviors were perceived as simple. In addition, at low levels of contextual stability, more complex behaviors were less likely to show automaticity than simpler behaviors, while at the highest levels of contextual stability, more complex behaviors were more likely to show greater automaticity than simpler behaviors (Figure 3, right panel). Frequency did not interact with behavioral complexity or contextual stability to predict high behavioral automaticity. Including interactive effects in the model significantly improved fit over the model including only main effects, χ^2^ (4, *N* = 459) = 31.61, *p* < 0.001.

**TABLE 4:** Results of Model 1: frequency, contextual stability, and rewards as predictors of habit strength, moderated by behavioral complexity.

**Predictor variable**	**Unstandardized *B***	***SE***	**Standardized β**	***p***
**Model 1a (Main effects only)**				
Frequency	0.039	0.002	2.856	< 0.001^***^
Complexity	–0.006	0.003	–0.278	0.026^*^
Rewards	0.005	0.002	0.278	0.008^∗∗^
Contextual stability	0.020	0.003	0.866	< 0.001^***^
Age	–0.012	0.003	–0.539	< 0.001^***^
Male	0.0004	0.002	0.041	0.794
**Model 1b (Including interactive effects)**				
Frequency × Complexity	–0.00004	0.0001	–0.214	0.380
Frequency × Contextual stability	–0.00003	0.0001	–0.16	0.666
Complexity × Rewards	0.0002	0.0001	0.813	0.019^*^
Complexity × Contextual stability	0.0005	0.0001	2.176	< 0.001^***^

Model 1a tested only the main effects; Model 1b included interactive effects alongside the previously tested main effects. Both models included random effects of behavior and participant, with behaviors nested within participant. ^*^p < 0.05. ^∗∗^p < 0.01. ^∗∗∗^p < 0.001.

##### Individual behaviors

**FIGURE 4:**
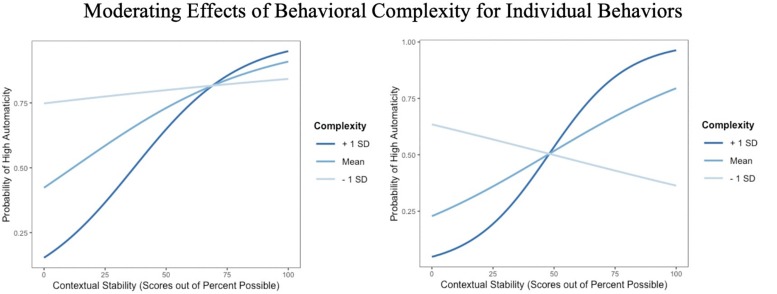
**(Left)** Probability of high automaticity for *handwashing* as a function of the stability of the context in which one does the behavior, moderated by behavioral complexity; lines curve due to the logistic analysis. **(Right)** Probability of high automaticity for *smoking* as a function of the stability of the context in which one does the behavior, moderated by behavioral complexity; lines curve due to the logistic analysis.

Model 1 was also run individually for the four behaviors that were rated in all three clusters: exercise, handwashing, smoking, and medication adherence (Table 5). Of these four behaviors, exercise was, on average, rated the most complex and handwashing was rated the simplest; exercise was also rated on average the most complex across the full sample of 25 behaviors, and handwashing was rated among the simplest (second only to sitting). Results for these behaviors generally showed parallel patterns to the multilevel model, with some exceptions. Behavioral frequency, contextual stability, and rewards each predicted high automaticity for all four control behaviors, with the exception that rewards did not predict automaticity for smoking. Perceived behavioral complexity predicted high automaticity only for exercise and medication adherence. Rewards did not interact with perceived complexity to predict automaticity for any of the behaviors, but contextual stability interacted with complexity to predict high automaticity for handwashing, and a similar trend emerged for smoking. When handwashing was perceived as complex, contextual stability was positively associated with high automaticity, but when handwashing was perceived as simple, the predictive value of contextual stability on automaticity was reduced (Figure 4, left panel). When smoking was perceived as complex, contextual stability was positively associated with high automaticity, but when smoking was perceived as simple, contextual stability was negatively associated with automaticity (Figure 4, right panel). When the interaction between frequency and context was included in the model, this effect was no longer significant for smoking. Nevertheless, the frequency and context interaction did not significantly predict automaticity.

**TABLE 5:** Results of Model 1 by individual behaviors: frequency, contextual stability, and rewards as predictors of habit strength, moderated by behavioral complexity.

***Behavior* Predictor variable**	**Unstandardized *B***	***SE***	**Standardized β**	***p***
**Exercise**				
Frequency	0.057	0.010	1.188	< 0.001^***^
Complexity	0.040	0.012	0.627	< 0.001^***^
Contextual stability	0.041	0.013	0.878	0.001^∗∗^
Rewards	0.010	0.008	0.267	0.207
Male	0.002	0.003	0.100	0.561
Age	–0.014	0.008	–0.305	0.111
Frequency × Complexity	0.001	0.001	0.203	0.539
Frequency × Contextual stability	0.001	0.001	0.270	0.457
Complexity × Contextual stability	–0.0004	0.001	–0.163	0.549
Complexity × Rewards	–0.0001	0.001	–0.022	0.920
**Handwashing**				
Frequency	0.023	0.006	0.450	< 0.001^***^
Complexity	0.002	0.007	0.053	0.730
Contextual stability	0.023	0.007	0.418	0.001^∗∗^
Rewards	0.011	0.004	0.334	0.009^∗∗^
Male	–0.003	0.003	–0.131	0.299
Age	–0.007	0.006	–0.157	0.198
Frequency × Complexity	–0.0002	0.0003	–0.104	0.434
Frequency × Contextual stability	–0.0002	0.0004	–0.094	0.495
Complexity × Contextual stability	0.001	0.0005	0.360	0.041^*^
Complexity × Rewards	0.0005	0.0003	0.299	0.091
**Smoking**				
Frequency	0.042	0.009	1.505	< 0.001^***^
Complexity	0.036	0.012	0.954	0.003^∗∗^
Contextual stability	0.013	0.013	0.262	0.334
Rewards	–0.002	0.010	–0.038	0.875
Male	–0.003	0.005	–0.128	0.582
Age	–0.019	0.012	–0.389	0.108
Frequency × Complexity	–0.001	0.0004	–0.524	0.201
Frequency × Contextual stability	–0.0002	0.0004	–0.148	0.608
Complexity × Contextual stability	0.001	0.0008	0.771	0.080
Complexity × Rewards	0.0004	0.0006	0.250	0.526
**Medication adherence**				
Frequency	0.017	0.006	0.490	0.005^∗∗^
Complexity	0.015	0.008	0.342	0.055
Contextual stability	0.048	0.011	0.918	< 0.001^***^
Rewards	0.020	0.005	0.673	< 0.001^***^
Male	–0.0001	0.003	–0.003	0.985
Age	0.0001	0.0001	0.146	0.340
Frequency × Complexity	0.0002	0.0003	0.126	0.522
Frequency × Contextual stability	–0.0003	0.0004	–0.151	0.499
Complexity × Contextual stability	–0.0002	0.0006	–0.086	0.730
Complexity × Rewards	0.0004	0.0002	0.302	0.089

In all models, interactions and main effects were entered separately. ^*^p < 0.05. ^∗∗^p < 0.01. ^∗∗∗^p < 0.001.

#### Model 2

**FIGURE 5:**
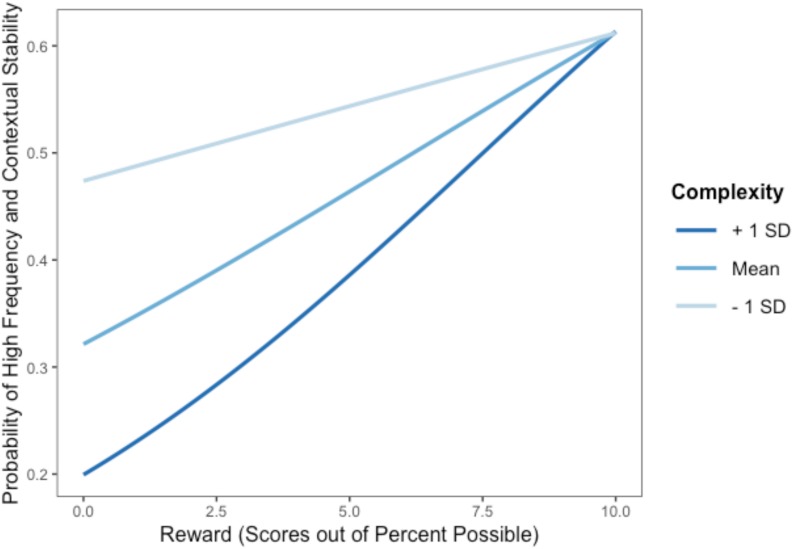
Probability of high habit strength as a function of perceived rewards, moderated by complexity; lines curve due to the logistic analysis.

Model 2 (Figure 2) aimed to replicate findings of Model 1, using the BF × CS measurement of habit strength in place of automaticity. As Model 1 used a binomial logistic distribution, the BF × CS variable was also stratified into ‘high’ and ‘low’ using a median split in the interests of replication. In Model 2, rewards again were associated with high habit strength, and complexity was negatively associated with habit strength (Table 6). Complexity further interacted with rewards to predict habit strength, following the same patterns found in Model 1; when behaviors were perceived as complex, rewards were stronger predictors of high habit strength (Figure 5), compared to when behaviors were seen as simple. Including the interaction term significantly improved the fit of the model, χ^2^ (1, *N* = 459) = 23.47, *p* < 0.001.

**TABLE 6:** Results of Model 2: rewards as associated with of habit strength (BF × CS), moderated by behavioral complexity.

**Predictor variable**	**Unstandardized *B***	***SE***	**Standardized β**	***p***
**Model 2a (Main effects only)**				
Rewards	0.108	0.013	0.660	< 0.001^***^
Complexity	–0.010	0.002	–0.491	< 0.001^***^
Age	–0.001	0.002	–0.028	0.774
Male	–0.0003	0.001	–0.029	0.769
**Model 1b (Including interactive effects)**				
Complexity × Rewards	0.003	0.001	1.295	< 0.001^***^

*Model 2a tested only the main effects; Model 2b included interactive effects alongside the previously tested main effects. Both models included random effects of behavior and participant, with behaviors nested within participant. ^∗∗∗^p < 0.001.*

### *Post hoc* Analyses

Preliminary analyses suggested that unhealthy behaviors were more automatic than healthy behaviors. A mediation analysis evaluated whether behavioral complexity was confounded with unhealthiness of behavior in the present study. A significant mediation effect emerged (ACME = 0.019, *p* < 0.001), with behavioral complexity accounting for 42.6% of the association between unhealthy behavior and automaticity. Unhealthiness of the behavior was no longer associated with automaticity when behavioral complexity was accounted for (β = 0.122, *p* = 0.18), suggesting complete mediation.

Given that rewards have been predicted to promote habit strength by promoting intention to engage in the behavior, an additional mediation analysis tested if intention explained the effect of rewards in Model 1; it did not (ACME = −0.0001, *p* = 0.084).

Finally, a model evaluated the predictive validity of automaticity on behavior enactment in our sample. As behavior enactment was bimodally distributed around the extremes, a logistic analysis was again used. Results revealed that automaticity significantly predicted behavior above and beyond the effects of intention and perceived behavioral control alone, χ^2^(1, *N* = 459) = 595.88, *p* < 0.001.

## Discussion

The present study confirmed that, across 25 behaviors, behavioral frequency, contextual stability, and rewards were each associated with behavioral automaticity. It additionally established that complexity of the behavior predicts automaticity and interacts with both contextual stability and rewards, thus providing insights to the role of behavioral complexity in habitual processes (Figure 3). Together, these findings provide clarity regarding the components of habits across multiple domains of behavior.

The interactive effects of complexity on the influence of rewards and contextual stability on automaticity explains the ways in which experiences of a behavior lend to non-effortful control. Rewards are associated with positive attitudes and intentions, and they may provide utilitarian function in promoting engagement in beneficial behaviors (e.g., even beyond the influence of intentional processes; Diamond and Loewy, 1991). Johnson et al. (2019) maintained that rewards impact habit strength by promoting intention to perform the behavior in the future, and Boynton (2005) found that executing complex behaviors (e.g., studying for an exam) is more dependent on intention than simpler behaviors (e.g., using a seatbelt). In line with this previous literature, we had expected that rewards would positively predict behavioral automaticity, and that this association would be strengthened with more complex behaviors. Both patterns appeared, when using either automaticity and the BF × CS interaction as measures of habit strength. Thus, regardless of whether one considers habit as a function of automaticity or as a function of frequency and contextual stability, perceptions of rewards and complexity are important components of habit strength.

Still, *post hoc* analyses found no significant mediation effect in which the influence of rewards on automaticity was explained by greater intention for rewarded behaviors. These findings cast doubt on an association of rewards and habit strength solely through intention, but are, nonetheless, in line with other recent research. For example, Phillips et al. (2016) found that rewards predicted exercise behavior through intention for behavior instigators, but not for behavior maintainers; possibly, in the habit formation process, intention increases initially, but diminishes as habits develop. Due to the cross-sectional nature of this study, the present research was not able to give a full picture of rewards in behavior for initiators compared to maintainers. Judah et al. (2018) also found only inconsistent support that rewards predicted habit development through increased behavioral repetition; rather, rewards impacted habit strength by strengthening the association between doing a behavior and habit development.

The present study did not test a moderation association between rewards, behavioral frequency, and habit strength, but if complex behaviors are executed less frequently due to the number of steps and time involved in doing these behaviors, rewards may be more important for habit development for complex behaviors than simple, frequently executed behaviors by strengthening the effect of few repetitions. Additionally, Lally et al. (2010) found a logarithmic function of habit development over frequency; plausibly, rewards might drive this pattern by providing diminishing returns with each repetition. Indeed, the operant conditioning literature has established that continuous reinforcement is not as effective for long-term behavior change as variable reinforcement (Guttman, 1953), and Stawarz et al. (2015) found that although rewards effectively promoted behavior, *automaticity* development was hindered. Thus, simple behaviors that can easily be executed may not benefit as strongly as complex behaviors from the presence of rewards due to a function of diminishing returns.

Thus, while TPB approaches have argued that rewards impact behavior by promoting positive attitudes toward a behavior, which then increases intention to engage in the behavior, the present research confirms that rewards are also instrumental in non-intentional behavioral processes. In the case of positive, healthy behaviors, this reward-based process can promote self-regulation by transferring control past the limits of intention and yielding long-term behavior change (Lally and Gardner, 2013). Yet, in the case of unhealthy or negative behaviors, rewards have the potential to circumvent self-regulation efforts (Johnson et al., 2019). The present findings support the need for a more nuanced understanding of the mechanisms through which rewards yield behavior in habits and other forms of non-effortful control.

It was hypothesized that complexity and contextual stability would interact to predict automaticity such that contextual stability would be a stronger predictor of automaticity when complexity is low. The results *did* reveal this pattern, which lends support to the argument that simple behaviors might be executed easily in multiple contexts, such that multiple cues might come to cue the same behavior. If habits are understood as the impulse toward a given behavior when an individual encounters a particular cue (Lally and Gardner, 2013), measurement of simple behavioral habits using self-report measures might not target a single habit, but rather multiple habits related to executing the same behavior. As the present study did not directly measure the specific cues that trigger habitual behaviors for each individual, this explanation cannot be further substantiated. An alternative argument might posit that while complex and simple habits have the potential to be triggered by a single environmental cue, complex behaviors require more complex cues that depend on multiple broader aspects of the overall context, while simpler habits can be initiated in response to a simple cue that can exist in multiple contexts. For instance, an individual’s exercise habit might be cued when they see their sneakers by the door, but only after work and when the weather is fair, while the same individual’s seatbelt habit might be cued every time they sit in a car, regardless of time of day or weather conditions. Such experiences have been reported qualitatively in previous research (Lally et al., 2011).

An unexpected interaction between contextual stability and complexity also appeared, such that when contextual stability was high, more complex behaviors were associated with greater automaticity compared to simpler behaviors. This finding appears counter-intuitive; we had no reason to expect that more complex behaviors become *more* automatic than simple behaviors when both the simple and complex behaviors are performed in stable contexts. The interaction found in this study may be an artifact of using self-report measures of automaticity across such a spectrum of behaviors; the validity of asking individuals the extent to which they enact a behavior ‘without awareness’ has been previously questioned (Hagger et al., 2015). It is possible – perhaps even likely – that participants scored the extent to which they executed behaviors automatically based on what they considered was automatic for that *particular* behavior, rather than across behaviors. Doing so may have yielded different criteria by which the varying behaviors were rated as automatic. For instance, we hypothesized that contextual stability would be a stronger predictor for complex behaviors rather than simple behaviors as simple behaviors could be easily executed in multiple contexts, leading to automaticity across contexts. Our participants may have been using a similar lay theory; thus, when considering simple behaviors executed only in a particular context, they may have considered these behaviors to be less automatic *because* of their situational dependence, expecting that truly automatic simple behaviors would be executed regardless of context. Previous literature has shown, for example, that social smokers are less likely than those who smoke in multiple contexts to identify as smokers or to consider their behavior a ‘personal addiction’ (Moran et al., 2004), but may nevertheless reflect physiological addiction (DiFranza and Wellman, 2005).

The findings of this study largely supported the hypotheses, but other results were surprising. No effect of age was hypothesized, but age was found to be *negatively* associated with automaticity in the first model. It is possible this finding was driven by the choice of behaviors assessed in this study; alcohol consumption has been shown to peak in young adulthood (Britton et al., 2015), and several behaviors assessed in the present study are dependent on phone or internet use (such as texting and driving and IT use), which are associated with younger age (Andone et al., 2016; Neves et al., 2018).

In the first model, an interaction between behavioral frequency and complexity was predicted, such that when complexity was high, frequency would be a weaker predictor of habit strength, but no interaction was found. The present findings would suggest that the association between behavioral frequency and complexity as predictors of habit strength is purely additive. To our knowledge, the present study is the first to examine an interaction between frequency and complexity, and the present findings might support the interpretation of Verplanken’s (2006) results as an additive association. While individuals in the simple task condition had higher habit strength than those in the complex task condition when frequency was held constant, perhaps the simple task condition *started* with higher habit strength due to the low levels of complexity.

Further, the BF × CS interaction did not significantly predict automaticity after accounting for the main effects of frequency and contextual stability. This null effect is perhaps surprising given that BF × CS is frequently used as a proxy for habit strength. Taken with the finding that contextual stability is less associated with automaticity when complexity is low rather than high, these results may suggest a need to better understand contextual stability in habits. Frequency and contextual stability may have additive rather than interactive associations with habit-related automaticity. Yet, rewards and complexity were similarly associated with the BF × CS interaction as with automaticity; regardless of whether one considers habits as automaticity or as patterns of behavior, these components of habit hold constant. Thus the present findings appear to be relatively robust.

While the multilevel model assessed factors associated with automaticity across behaviors while accounting for random effects of individuals, the following single-level models compared individuals on a single behavior. These single-level models examining individual behaviors (see Table 5) provide insights into the components of habit strength when behavioral characteristics are held consistent. For instance, frequency was associated with automaticity for each individual behavior assessed, but rewards were associated with automaticity only for the health promotion behaviors of exercise, handwashing, and medication adherence, and not for the health risk behavior of smoking. Thus, the prominence of frequency as a factor of habit is maintained, and rewards are important factors for behavioral automaticity, but further behavioral moderators may need to be considered.

In addition, the single-level models provide particular insights to the role of *perceived* complexity, as examining single behaviors at a time holds the objective complexity constant. When decomposing the first model to test the influence of each behavioral frequency, contextual stability, perceived rewards, and behavioral complexity on automaticity for individual behaviors, the patterns found across the full spectrum behaviors did always not hold consistent. Some associations with automaticity for individual behaviors were surprising; for each exercise, smoking, and medication adherence, perceived complexity was *positively* associated with high automaticity. Further, participants tended to rate exercise as more complex (*M* = 64.06) than handwashing (*M* = 32.01), yet, despite the finding that rewards were a stronger predictor for complex, rather than simple, behaviors when assessing all behaviors, rewards were only a significantly associated with automaticity for handwashing and not exercise. These findings further support the need to better understand the factors that yield perceptions of behavioral complexity for different behaviors; for instance, individuals who are required to take multiple daily medications may perceive medication adherence as complex, but have stronger habits for medication adherence than someone who only takes only one pill daily for a relatively minor condition. An individual who exercises moderately by jogging a few times a week may view exercise as relatively non-complex, while a ‘gym rat’ who devotes a significant amount of time to daily exercise may have an elaborate exercise routine. The Dunning-Kruger effect may also have played a role in the present findings, as individuals who engage more in particular behaviors may come to understand the complexities involved with that behavior, compared to those who have only had passing experiences with a behavior (Dunning, 2011). Thus, the individual behavioral models may point to additional moderators for future research examining habits across behaviors, such as health importance or knowledge of the behaviors. Further analyses with objective measures of complexity might also be compared to the present findings to confirm the influence of *perceived* complexity as compared to objective complexity. Given the theoretical non-reasoned pathways of habitual control, differential influences of perceived and objective complexity would be particularly interesting.

This study further supported the validity of a five-item version of Boynton’s (2005) behavioral complexity scale using a large sample assessing a diverse span of behaviors. Future research might draw on this short, easily administered scale to assess the extent to which perceived behavioral complexity predicts behavior outcomes. Unfortunately, the other two new scales assessed by this study were not as well supported. Low’s (2016) measure of contextual stability showed good reliability but was found to load onto two factors, rather than a single factor. The presence of two factors in this scale might call to question the structure of a behavioral ‘context.’ Previous descriptions of context in the Principle of Compatibility have called for consideration of broad contextual factors on equal levels of generality or specificity (Ajzen, 1988), but have not detailed key facets of such contexts. Examination of the two factors that appeared in this study reveals a factor loading on the *physical* environment as well as a factor loading on the *social* environment. Future research might assess if physical and social contexts differentially influence behavioral predictors. Regardless, the scale of contextual stability did not fit particularly well on a two-factor model. The items of this scale could be adjusted and re-assessed to examine if a better-fitting two-factor structure emerges. Following such adjustments, this scale has the potential to be a valid assessment of contextual stability that provides a broader assessment than extant measures. The rewards scale showed remarkably poor reliability and validity, which may suggest this scale does not generalize to all behaviors. Different measures of rewards should be used and evaluated in future research.

### Limitations and Future Directions

The findings of this study are limited by measurement validity. Several variables were assessed with a single item, and the contextual stability scale did not load well onto the expected one-factor model. Issues of measurement validity are evident in our results by the convergence of our models (Model 1 converged at gradient 0.100, while Model 2 converged at gradient 0.0004), and by the existence of standardized effect sizes greater than 1, which were not accounted for by multicollinearity. In light of considering these issues, the current findings should be interpreted with caution, and future analyses should aim to substantiate the findings of the present study with improved measures. In particular, the use of new measures for rewards and frequency would be particularly apt, given that each of these variables were measured with a single item in the present study. In addition, this study examined factors that have been theorized to lead to habit development, but only using cross-sectional methods; thus, each factor was shown to be associated with habit strength, but not explicitly to be involved with the process of habit development. Longitudinal replications are needed to support our findings.

Also, habits were measured using the SRBAI, which represents one of the shortest, validated measures tapping automaticity in habit strength. Despite the practical strengths of this measure, the SRBAI does not directly examine habits as a function of cue-behavior association, which is an important aspect of habits (Wood and Neal, 2016). As a result, the SRBAI may potentially fail to differentiate between habits and other non-learned forms of automaticity (Gardner, 2015). Regardless, findings from the second model in the present study reveal that similar patterns emerge when using alternative measurements of habit strength. No measure yet adequately taps all three dimensions of frequency, automaticity, and cue-behavior association, but as such measures are developed, findings from the present study might be further replicated with these new measures. Further, one item of the SRBAI measures the extent to which a behavior is performed frequently; in the present study, this item overlaps with the predictor of frequency, and may account for the remarkably high association between frequency and automaticity, or for the null association between the BF × CS and automaticity, after accounting for the main effect of frequency. An association between frequency and automaticity is unsurprising and has been supported many times in the literature, but in order to more accurately assess the relative associations between each habit ‘ingredient’ and automaticity, alternative measures that do not directly tap frequency should be used in the future.

There are alternative ways the construct of ‘rewards’ might be considered. The rewards item used in the present study assessed rewards as a function of the extent to which an individual finds the behavior to be *pleasurable* – which can be thought of as an immediate, sensory experience (Judah et al., 2018). This approach draws on the conceptualization of rewards in animal learning models of habit (e.g., Broadbent et al., 2007). Other studies have also frequently examined rewards in habits by assessing *intrinsic motivation*, or the inclination to act because of inherent enjoyment of the behavior (e.g., Gardner and Lally, 2013; Phillips et al., 2016). Pleasure and intrinsic motivation have been shown to have similar patterns of influence on habit strength, suggesting that both may be valid ways of tapping the rewards pathway (Judah et al., 2018), but future research measuring rewards as intrinsic motivation may further substantiate our findings. Rewards might also be conceptualized as extrinsic rewards: that is, as a reinforcement external to the behavior. Previous literature has suggested that external rewards might in fact undermine habit development (Wood and Neal, 2016), but future research might assess if complexity impacts this association as well.

Given that behavioral complexity and healthiness of behaviors were confounded in the present study, a different sampling of behaviors may yield a more complete picture of habits in healthy and unhealthy behaviors. Engagement in unhealthy behavior may also be influenced by low levels of social desirability and other factors specific to undesired behaviors that were not assessed in this research. Further studies might assess the different pathways by which healthy and unhealthy habits develop, controlling for complexity in order to understand the influence of these other factors. That said, the current sample of behaviors was drawn largely from the habits literature; present findings suggest that commonly studied health promotion and health risk behaviors may have different associations with habit in part *due* to varying levels of complexity, which substantiates the need to understand behavioral complexity in habits. Participants in the present study reported also consistently high levels of intention and perceived behavioral control, even for unhealthy behaviors; as such, findings may not be generalizable to unintended habits. Future research may wish to compare the factors associated with intended as compared to non-intended habits.

This study focused primarily on the components of habit *development*; future research might assess the influence of complexity on habit *disruption*. Previous research has often focused on habit disruption through changing contexts (e.g., Wood et al., 2005; Verplanken et al., 2008). If contextual stability is a stronger predictor of habit strength for complex, rather than simple behaviors, this approach might be more effective for changing complex behaviors and less effective for simpler behaviors such as the health-risk behaviors assessed in this study. Given the influence of habits on behavior beyond that of intention, understanding the role of complexity in disruption of unwanted habits would improve efforts at behavior change in negative or health-risk behaviors.

## Conclusions

In sum, this study confirms that each of the three ‘ingredients’ of habit development proposed by Wood and Neal (2016) – behavioral frequency, contextual stability, and rewards – are independently associated with automaticity across a broad spectrum of behaviors, and that complexity of the behavior often influences these associations. Perceived behavioral complexity appears to strengthen the associations of rewards and contextual stability on habit strength, and thus behavioral complexity is an important factor in mapping habitual processes and is worthy of future investigations to better understand it.

## Data Availability

The datasets generated for this study are available on request to the corresponding author.

## Ethics Statement

This study was approved by the University of Connecticut Institutional Review Board (IRB) on August 9th, 2018 (Protocol #X18-095). This study was exempt from collecting written consent; the procedures were deemed to be low risk, and collecting signed consent would increase the risk level as signed consent would constitute the only identifiable information collected. Before the start of the study, participants were provided with an information sheet describing the details of the study. If participants agreed to the terms described, they were instructed to continue through to the full survey.

## Author Contributions

KM and BTJ conceptualized and designed the study. KM collected and organized the data, and further performed statistical analysis and developed the first draft of this manuscript under the guidance of BTJ. Both authors contributed to manuscript revision, and read and approved the submitted version.

## Conflict of Interest Statement

The authors declare that the research was conducted in the absence of any commercial or financial relationships that could be construed as a potential conflict of interest.

## Appendix

### Measures

#### Contextual Stability

When you [do behavior], how consistently do you do it… 0(Never the Same)… 10(Always the same)

(1) At the same time(s) of day?
(2) On the same day(s) of the week?
(3) For the same amount(s) of time per sitting?
(4) In the same particular way(s)?
(5) In the same location(s)?
(6) With the same people (who are also doing it)?
(7) Around the same people (who are NOT doing it)?
(8) Using the same object(s) or tool(s)?

#### Rewards

When you [do behavior] how does it feel? 0(Not at all)…… . . 10(Extremely)

(1) Pleasurable?

#### Behavioral Complexity

1(Not at All)… . . 7(A Great Deal)

(1) In general, how challenging is [behavior] for the average person?
(2) How much attention does the average person need to give to [behavior] to do it well?
(3) How complex is [behavior]?
(4) How much planning is involved before [behavior]?
(5) On average, how much time does it take to [behavior]?

### Behavior Definitions

#### Exercise

By exercise, I mean engaging in physical behavior for 30 min or more that elevates your heart rate.

#### Handwashing

By handwashing, I mean washing your hands in any context.

#### Smoking

By smoking, I mean using cigarettes to smoke tobacco.

#### Taking Medication

By taking medication, I mean taking medication that has been prescribed to you by a healthcare professional.

#### Fruit and Vegetable Consumption

By eating fruits and vegetables I mean any time you eat fruits or vegetables, not necessarily at the same time.

#### Unhealthy Snacking

By unhealthy snacking I mean eating foods high in fat or sugar not at meal times.

#### Alcohol Consumption

By drinking alcohol, I mean drinking at least one unit of alcohol (approximately one measure of spirits, half a glass of wine, or half a pint of beer).

#### Internet Use

By internet use, I mean accessing the internet through computers, phones, or tablets for either leisure or work use.

#### Seafood Consumption

By eating seafood, I mean eating fish or shellfish.

#### Food Safety Practices

By food safety practices, I mean handling and treating food in such a way as to reduce the risk of getting sick from food, such as washing hands and surfaces when handling food, keeping food at the “correct” temperature, and avoiding unsafe foods.

#### Playing Video Games

By playing video games, I mean playing games on a computer or console.

#### Active Commuting

By active commuting, I mean traveling to and from work or school by a means that requires some physical activity on your part, such as walking, biking, or using public transport.

#### IT Use

By IT use, I mean using technology to save, receive, or send information.

#### Sunscreen Use

By sunscreen use, I mean applying a product with SPF protection to your skin.

#### Sitting

By sitting, I mean sitting in any context, such as in a car or on a bus, or at work/home/or school.

#### Flossing

For the following questions, I will ask you about your feelings and behaviors regarding flossing. By flossing, I mean using dental floss to floss your teeth.

#### Recycling

By recycling, I mean putting recyclable materials in recycling receptacles.

#### Playing Music

By playing music, I mean performing music by playing a musical instrument and/or by singing.

#### Car Use

By car use, I mean that when you need to use transportation, you drive a car.

#### Depositing Savings

By depositing savings, I mean putting money in a dedicated savings account.

#### Condom Use

By condom use, I mean using a condom while engaging in sexual activity.

#### Negative Self-Thoughts

By negative self-thoughts, I mean negative thoughts you have about yourself.

#### Sugary Drink Consumption

By drinking sugary drinks, I mean drinking beverages that are high in sugar, such as soda, energy drinks, or juice.

#### Phone Checking

By checking your phone, I mean checking your phone for notifications with or without a notification alert.

#### Texting and Driving

By texting and driving, I mean reading and sending texts and/or instant messages while driving.

## References

[B1] Ajzen I. (1988). *Attitudes, Personality, and Behavior.* New York, NY: McGraw-Hill Education.

[B2] Ajzen I. (2002). *Constructing a TPB Questionnaire: Conceptual and Methodological Considerations.* Available at: https://pdfs.semanticscholar.org/0574/b20bd58130dd5a961f1a2db10fd1fcbae95d.pdf (accessed May, 2018).

[B3] Ajzen I. Fishbein M. (2005). “The influence of attitudes on behavior,” in *The Handbook of Attitudes*, eds Albarracín D. Johnson B. T. Zanna M. P. (New York: Psychology Press), 173–221.

[B4] Andone I. Błaszkiewicz K. Eibes M. Trendafilov B. Montag C. Markowetz A. (2016). “How Age and Gender Affect Smartphone Usage,” in *Proceedings of the 2016 ACM International Joint Conference on Pervasive and Ubiquitous Computing*, (New York, NY: Association for Computing Machinery).

[B5] Bates D. Maechler M. Bolker B. Walker S. (2015). Fitting Linear Mixed-Effects Models Using lme4. *J. Stat. Softw.* 67 1–48. 10.18637/jss.v067.i01

[B6] Boynton M. (2005). *The Role of Complexity in The Prediction and Modeling of Automatic and Deliberative Behaviors.* (Masters thesis).Storrs, CT: University of Connecticut.

[B7] Britton A. Ben-Shlomo Y. Benzeval M. Kuh D. Bell S. (2015). Life course trajectories of alcohol consumption in the United Kingdom using longitudinal data from nine cohort studies. *BMC Med.* 13:47. 10.1186/s12916-015-0273-z 25858476 PMC4351673

[B8] Broadbent N. J. Squire L. R. Clark R. E. (2007). Rats depend on habit memory for discrimination learning and retention. *Lear. Mem.* 14 145–151. 10.1101/lm.455607 17351137 PMC1838555

[B9] Cerasoli C. P. Nicklin J. M. Ford M. T. (2014). Intrinsic motivation and extrinsic incentives jointly predict performance: a 40-year meta-analysis. *Psychol. Bull.* 140 980–1008. 10.1037/a0035661 24491020

[B10] Cohen P. Cohen J. Aiken L. S. West S. G. (1999). The problem of units and the circumstance for POMP. *Multivariate Behav. Res.* 34 315–346. 10.1207/s15327906mbr3403_2

[B11] de Vet E. Oenema A. Brug J. (2011). More or better: do the number and specificity of implementation intentions matter in increasing physical activity? *Psychol. Sport Exerc.* 12 471–477. 10.1016/j.psychsport.2011.02.008

[B12] de Wit S. Dickinson A. (2009). Associative theories of goal-directed behaviour: a case for animal–human translational models. *Psychol. Res.* 73 463–476. 10.1007/s00426-009-0230-6 19350272 PMC2694930

[B13] Diamond W. D. Loewy B. Z. (1991). Effects of Probabilistic Rewards on Recycling Attitudes and Behavior 1. *J. Appl. Soc. Psychol.* 21 1590–1607. 10.1111/j.1559-1816.1991.tb00489.x

[B14] DiFranza J. R. Wellman R. J. (2005). A sensitization—homeostasis model of nicotine craving, withdrawal, and tolerance: integrating the clinical and basic science literature. *Nicotine Tob. Res.* 7 9–26. 10.1080/14622200412331328538 15804674

[B15] Diggle P. J. Liang K. Y. Zeger S. L. (1995). *Analysis of Longitudinal Data.* New York, NY: Oxford University Press.

[B16] Dong Y. Peng C. Y. J. (2013). Principled missing data methods for researchers. *SpringerPlus* 2 222–239. 23853744 10.1186/2193-1801-2-222 PMC3701793

[B17] Dunning D. (2011). The Dunning–Kruger effect: on being ignorant of one’s own ignorance. *Adv. Exp. Soc. Psychol.* 44 247–296. 10.1016/b978-0-12-385522-0.00005-6

[B18] Fatseas M. Serre F. Alexandre J. M. Debrabant R. Auriacombe M. Swendsen J. (2015). Craving and substance use among patients with alcohol, tobacco, cannabis or heroin addiction: a comparison of substance-and person-specific cues. *Addiction* 110 1035–1042. 10.1111/add.12882 25688760

[B19] Fishbein M. Ajzen I. (1975). *Belief, Attitude, Intention and Behavior: An introduction to Theory and Research.* Boston, MA: Addison-Wesley.

[B20] Fishbein M. Ajzen I. (2011). *Predicting and Changing Behavior: The Reasoned Action Approach.* New York, NY: Psychology Press.

[B21] Gardner B. (2015). A review and analysis of the use of ‘habit’ in understanding, predicting and influencing health-related behaviour. *Health Psychol.Rev.* 9 277–295. 10.1080/17437199.2013.876238 25207647 PMC4566897

[B22] Gardner B. Abraham C. Lally P. de Bruijn G.-J. (2012). Towards parsimony in habit measurement: testing the convergent and predictive validity of an automaticity subscale of the Self-Report Habit Index. *Int. J. Behav. Nutr. Phys. Act.* 9 1–12. 10.1186/1479-5868-9-102 22935297 PMC3552971

[B23] Gardner B. Lally P. (2013). Does intrinsic motivation strengthen physical activity habit? Modeling relationships between self-determination, past behaviour, and habit strength. *J. Behav. Med.* 36 488–497. 10.1007/s10865-012-9442-0 22760451

[B24] Gilpin E. A. Lee L. Pierce J. P. (2004). Does adolescent perception of difficulty in getting cigarettes deter experimentation? *Prev. Med.* 38 485–491. 10.1016/j.ypmed.2003.12.001 15020183

[B25] Guttman N. (1953). Operant conditioning, extinction, and periodic reinforcement in relation to concentration of sucrose used as reinforcing agent. *J. Exp. Psychol.* 46 213–224. 10.1037/h0061893 13109117

[B26] Hagger M. S. Chatzisarantis N. L. Biddle S. J. (2002). A meta-analytic review of the theories of reasoned action and planned behavior in physical activity: predictive validity and the contribution of additional variables. *J. Sport Exerc. psychol.* 24 3–32. 10.1123/jsep.24.1.3

[B27] Hagger M. S. Rebar A. L. Mullan B. Lipp O. V. Chatzisarantis N. L. (2015). The subjective experience of habit captured by self-report indexes may lead to inaccuracies in the measurement of habitual action. *Health Psychol. Rev.* 9 296–302. 10.1080/17437199.2014.959728 25189762

[B28] Huff C. Tingley D. (2015). Who are these people? Evaluating the demographic characteristics and political preferences of MTurk survey respondents. *Res. Polit.* 2 1–12.

[B29] Ibm Corp. (2017). *IBM SPSS Statistics for Windows, Version 25.0.* Armonk, NY: IBM Corp.

[B30] Johnson B. T. Landrum A. McCloskey K. (2019). “Attitude scholarship in the 21st century: Accomplishments, challenges, and gaps,” in *The Handbook of Attitudes*, Vol. 1 (2nd Edn), eds Albarracín D. Johnson B. T. (New York, NY: Francis & Taylor), 625–649.

[B31] Judah G. Gardner B. Kenward M. G. DeStavola B. Aunger R. (2018). Exploratory study of the impact of perceived reward on habit formation. *BMC Psychol.* 6:62. 10.1186/s40359-018-0270-z 30572936 PMC6302524

[B32] Lally P. Gardner B. (2013). Promoting habit formation. *Health Psychol. Rev.* 7 S137–S158.

[B33] Lally P. Van Jaarsveld C. H. Potts H. W. Wardle J. (2010). How are habits formed: modelling habit formation in the real world. *Eur. J. Soc. Psychol.* 40 998–1009. 10.1002/ejsp.674

[B34] Lally P. Wardle J. Gardner B. (2011). Experiences of habit formation: a qualitative study. *Psychol. Health Med.* 16 484–489. 10.1080/13548506.2011.555774 21749245

[B35] Long J. A. (2018). *jtools: Analysis and Presentation of Social Scientific Data. R package Version 1.1.1.* https://cran.r-project.org/package=jtools

[B36] Low R. E. (2016). *Place in Habits and Habits in Place.* Available at: https://opencommons.uconn.edu/dissertations/1103 (accessed June 5, 2016).

[B37] McEachan R. R. C. Conner M. Taylor N. J. Lawton R. J. (2011). Prospective prediction of health-related behaviours with the theory of planned behaviour: a meta-analysis. *Health Psychol. Rev.* 5 97–144. 10.1080/08870446.2011.613995 21929476

[B38] McGinn A. P. Evenson K. R. Herring A. H. Huston S. L. Rodriguez D. A. (2007). Exploring associations between physical activity and perceived and objective measures of the built environment. *J. Urban Health* 84 162–184. 10.1007/s11524-006-9136-4 17273926 PMC2231636

[B39] Moran S. Wechsler H. Rigotti N. A. (2004). Social smoking among US college students. *Pediatrics* 114 1028–1034. 10.1542/peds.2003-0558-l 15466101

[B40] Morris T. P. White I. R. Royston P. (2014). Tuning multiple imputation by predictive mean matching and local residual draws. *BMC Med. Res. Methodol.* 14:75. 10.1186/1471-2288-14-75 24903709 PMC4051964

[B41] Neal D. T. Wood W. Quinn J. M. (2006). Habits—A repeat performance. *Curr. Dir. Psychol. Sci.* 15 198–202. 10.1111/j.1467-8721.2006.00435.x

[B42] Neves B. B. Fonseca J. R. Amaro F. Pasqualotti A. (2018). Social capital and Internet use in an age-comparative perspective with a focus on later life. *PLoS One* 13:e0192119. 10.1371/journal.pone.0192119 29481556 PMC5826529

[B43] Norman P. Cooper Y. (2011). The theory of planned behaviour and breast self-examination: assessing the impact of past behaviour, context stability and habit strength. *Psychol. Health* 26 1156–1172. 10.1080/08870446.2010.481718 21391130

[B44] Ouellette J. A. Wood W. (1998). Habit and intention in everyday life: the multiple processes by which past behavior predicts future behavior. *Psychol. Bull.* 124 54–74. 10.1037//0033-2909.124.1.54

[B45] Phillips L. A. Chamberland P. É Hekler E. B. Abrams J. Eisenberg M. H. (2016). Intrinsic rewards predict exercise via behavioral intentions for initiators but via habit strength for maintainers. *Sport Exerc. Perform. Psychol.* 5 352–364. 10.1037/spy0000071

[B46] R Core Team (2018). *R: A Language and Environment For Statistical Computing.* Vienna: R Foundation for Statistical Computing.

[B47] Revelle W. (2018). *Psych: Procedures for Personality and Psychological Research. R Package Version 1.8.4.* Evanston, Illinois: Northwestern University.

[B48] Rosseel Y. (2012). lavaan: an r package for structural equation modeling. *J. Stat. Softw.* 48 1–36. 10.3389/fpsyg.2014.01521 25601849 PMC4283449

[B49] Rothman A. J. Sheeran P. Wood W. (2009). ). Reflective and automatic processes in the initiation and maintenance of dietary change. *Ann. Behav. Med* 38(Suppl._1), s4–s17. 10.1007/s12160-009-9118-3 19787308

[B50] Stawarz K. Cox A. L. Blandford A. (2015). “Beyond self-tracking and reminders: designing smartphone apps that support habit formation,” in *Proceedings of The 33rd annual ACM Conference on Human Factors in Computing Systems*, (Seoul).

[B51] Tingley D. Yamamoto T. Hirose K. Keele L. Imai K. (2014). mediation: r package for causal mediation analysis. *J. Stat. Softw.* 59 1–38.26917999

[B52] van Buuren S. Groothuis-Oudshoorn K. (2011). mice: multivariate imputation by chained equations in r. *J. Stat. Softw.* 45 1–67.

[B53] Verhoeven A. A. Adriaanse M. A. De Ridder D. T. De Vet E. Fennis B. M. (2013). Less is more: the effect of multiple implementation intentions targeting unhealthy snacking habits. *Eur. J. Soc. Psychol.* 43 344–354. 10.1002/ejsp.1963

[B54] Verplanken B. (2006). Beyond frequency: habit as mental construct. *Br. J. Soc. Psychol.* 45 639–656. 10.1348/014466605x49122 16984725

[B55] Verplanken B. Orbell S. (2003). Reflections on past behavior: a self-report index of habit strength. *J. App. Soc. Psychol.* 33 1313–1330. 10.1111/j.1559-1816.2003.tb01951.x

[B56] Verplanken B. Walker I. Davis A. Jurasek M. (2008). Context change and travel mode choice: combining the habit discontinuity and self-activation hypotheses. *J. Env. Psychol.* 28 121–127. 10.1016/j.jenvp.2007.10.005

[B57] Webb T. L. Sheeran P. (2008). Mechanisms of implementation intention effects: the role of goal intentions, self-efficacy, and accessibility of plan components. *Br. J. Soc.Psychol.* 47 373–395. 10.1348/014466607x267010 18096108

[B58] Wiedemann A. U. Gardner B. Knoll N. Burkert S. (2014). Intrinsic rewards, fruit and vegetable consumption, and habit strength: a three-wave study testing the associative-cybernetic model. *Appl. Psychol. Health Well Being* 6 119–134. 10.1111/aphw.12020 24227692

[B59] Wood W. (2017). Habit in personality and social psychology. *Pers. Soc. Psychol. Rev* 21 389–403. 10.1177/1088868317720362 28737111

[B60] Wood W. Neal D. T. (2009). The habitual consumer. *J. f Consum.Psychol.* 19 579–592. 10.1016/j.jcps.2009.08.003

[B61] Wood W. Neal D. T. (2016). Healthy through habit: interventions for initiating & maintaining health behavior change. *Behav. Sci. Policy* 2 71–83. 10.1353/bsp.2016.0008

[B62] Wood W. Quinn J. M. Kashy D. A. (2002). Habits in everyday life: thought, emotion, and action. *J. Pers. Soc. Psychol.* 83 1281–1297. 10.1037//0022-3514.83.6.1281 12500811

[B63] Wood W. Tam L. Witt M. G. (2005). Changing circumstances, disrupting habits. *J. Pers. Soc. Psychol.* 88 918–933. 10.1037/0022-3514.88.6.918 15982113

[B64] Zeger S. L. Liang K. Y. Albert P. S. (1988). Models for longitudinal data: a generalized estimating equation approach. *Biometrics* 44 1049–1060. 3233245

