# Supplementary material for: Habits, Quick and Easy: Perceived Complexity Moderates the Associations of Contextual Stability and Rewards With Behavioral Automaticity
Source: Front Psychol. 2019 Jul 24;10:1556. doi: 10.3389/fpsyg.2019.01556 (PMC6667662; doi:10.3389/fpsyg.2019.01556)
Supplement: Supplementary file 1 [file Table_1.DOCX]

Supplemental Tables

Supplemental table. *Participant demographics.*

| Demographic | | n | % |
| --- | --- | --- | --- |
| Gender |  |  |  |
|  | Male | 260 | 56.6 |
|  | Female | 197 | 42.9 |
|  | Other | 2 | 0.4 |
| Age |  |  |  |
|  | 18-24 | 36 | 7.8 |
|  | 25-34 | 222 | 48.4 |
|  | 35-44 | 113 | 24.6 |
|  | 45-54 | 50 | 10.9 |
|  | 54-65 | 29 | 6.3 |
|  | 65+ | 9 | 2.0 |
| Income |  |  |  |
|  | Less than $20,000 | 46 | 10.0 |
|  | $20,000 to $34,999 | 92 | 20.0 |
|  | $35,000 to $49,999 | 108 | 23.5 |
|  | $50,000 to $74,999 | 118 | 25.7 |
|  | $75,000 to $99,999 | 55 | 12.0 |
|  | $100,000 to $149,999 | 26 | 5.7 |
|  | $150,000 to $199,999 | 13 | 2.8 |
|  | $200,000 to $299,999 | 1 | 0.2 |
| Residence |  |  |  |
|  | Urban | 161 | 35.1 |
|  | Suburban | 210 | 45.8 |
|  | Rural | 88 | 19.2 |
